# Newspaper framing of food poverty and insecurity on the island of Ireland

**DOI:** 10.1093/heapro/daaf206

**Published:** 2025-11-27

**Authors:** Claire Kerins, Páraic Kerrigan, Sinéad Furey, Aodheen McCartan, Colette Kelly, Tania Jahir, Elena Vaughan

**Affiliations:** Health Promotion Research Centre, School of Health Sciences, University of Galway, University Road, Galway H91 TK33, Ireland; Centre for Health Research Methodology, School of Nursing and Midwifery, University of Galway, University Road, Galway H91 TK33, Ireland; School of Information and Communication Studies, University College Dublin, Belfield, Dublin 4 D04 V1W8, Ireland; Department of Hospitality, Tourism and Events Management, Ulster University Business School, Ulster University, Cromore Road, Coleraine, Co. Londonderry BT52 1SA, United Kingdom; School of Communication and Media, Ulster University, York Street, Belfast, Co. Antrim BT15 1ED, United Kingdom; Health Promotion Research Centre, School of Health Sciences, University of Galway, University Road, Galway H91 TK33, Ireland; Health Promotion Research Centre, School of Health Sciences, University of Galway, University Road, Galway H91 TK33, Ireland; Health Promotion Research Centre, School of Health Sciences, University of Galway, University Road, Galway H91 TK33, Ireland

**Keywords:** food poverty, food insecurity, news media, newspaper, media framing, framing theory

## Abstract

Given that news media play key roles in shaping public and policy responses to food poverty and insecurity, this study analysed how newspapers frame these issues across the island of Ireland, comparing coverage between jurisdictions (Republic of Ireland and Northern Ireland) and newspaper types (national versus regional/local). Using LexisNexis and Irish Newspaper Archives, we searched for articles containing ‘food poverty’ or ‘food insecurity’ published between January 2018 and January 2023. We used Entman's framing theory to code articles for problem definitions, causal interpretations, solutions, moral evaluations, and social actors. Analysis of 80 articles from 14 newspapers revealed coverage peaked during school holiday periods (December and Summer), reinforcing episodic attention to ‘holiday hunger’. Although structural causes appeared in 66% of articles—including inadequate income, living costs, and welfare failures—proposed solutions were predominantly charitable (79%) rather than structural (39%), with food banks cited most frequently. National newspapers more frequently discussed structural causes and policy solutions, while regional publications focused on charitable responses. Articles predominantly featured voices from charities (88%) and government officials (50%), while only 10% incorporated voices from those experiencing food poverty and insecurity. This disconnect between acknowledged structural causes and proposed charitable solutions perpetuates normalization of food poverty and insecurity, obscuring state responsibility for ensuring the right to adequate food. Such framing impedes recognition that this issue in wealthy nations results from political choices requiring systemic reform, not charitable intervention.

Contribution to Health Promotion

- Newspapers across the island of Ireland acknowledge structural causes of food poverty and insecurity (66%) yet promote charitable solutions (79%)
- Coverage peaks during school holidays, treating the problem as seasonal ‘holiday hunger’ rather than persistent crisis
- Articles feature voices from charities (88%) and government officials (50%), while only 10% include affected individuals
- National newspapers more frequently discuss systemic drivers and policy solutions whereas local newspapers emphasize charitable responses
- Framing normalizes charitable responses, obscuring state responsibility for ensuring the right to adequate food
- Health promoters should work with media to reframe the issue as policy failure requiring systemic reform, not charity

## BACKGROUND

Food poverty and insecurity are complex phenomena with multiple dimensions. While often used interchangeably to describe the same health and social issue, these terms have distinct emphases in the literature. Food poverty is defined as ‘the insufficient economic access to an adequate quantity and quality of food to maintain a nutritionally satisfactory and socially acceptable diet’ (O'Connor *et al*. 2016, p.429). Food insecurity focuses on ‘the inability to consume an adequate quality or sufficient quantity of food in socially acceptable ways, or the uncertainty that one will be able to do so’ (Dowler and O'Connor 2012, p.45). Despite these definitional nuances, both concepts address fundamentally similar experiences of inadequate food access (O'Connor *et al*. 2016).

### Health and social impacts

Food poverty and insecurity represent violations of interconnected human rights, particularly the right to adequate food and the right to health (Dowler and O'Connor 2012, Campanera *et al*. 2023). The consequences are wide-ranging and severe for individuals and families. Meta-analyses demonstrate associations between food insecurity and micronutrient deficiencies (Lopes *et al*. 2023), metabolic and cardiovascular conditions including obesity (Carvajal-Aldaz *et al*. 2022, Salinas-Roca *et al*. 2022), mental health disorders including depression and anxiety (Pourmotabbed *et al*. 2020), and multimorbidity (Kantilafti *et al*. 2023). Among children, systematic reviews reveal associations with stunted growth (Patriota *et al*. 2024), cognitive impairment, behavioural problems, and poor academic performance (Rosen *et al*. 2024). Maternal food insecurity during pregnancy is associated with increased risk of gestational obesity (Nguyen *et al*. 2024), gestational diabetes mellitus, and poor maternal mental health outcomes (Bell *et al*. 2024).

These associations persist after adjustment for socioeconomic factors, suggesting mechanisms beyond poverty-mediated effects (Gallegos 2025), with evidence indicating a dose-response relationship where adverse health outcomes are observed even at marginal levels of food insecurity (Pérez-Escamilla *et al*. 2020). Beyond individual health impacts, food insecurity perpetuates cycles of poverty by reducing work productivity and increasing healthcare costs for both individuals and society (Swinburn *et al*. 2019, Himmelgreen *et al*. 2022). Recognition of these impacts has contributed to the inclusion of food security in the United Nations Sustainable Development Goals, with Goal 2 (Zero Hunger) targeting elimination of hunger by 2030 (United Nations General Assembly [UNGA] 2015).

### Prevalence and context on the island of Ireland

Food poverty and insecurity receive limited policy and research attention in high-income countries, where economic development may obscure their prevalence (Drew 2022, BMC Medicine Editorial 2023). Evidence demonstrates that household food insecurity affects between 8% and 20% of populations in developed countries (Pollard and Booth 2019). The island of Ireland comprises two distinct jurisdictions—the Republic of Ireland (ROI) and Northern Ireland (NI), which is part of the United Kingdom (UK)—each with separate health and social welfare systems, yet face comparable challenges regarding food poverty and insecurity. Food poverty affects an estimated 9% of the population in the ROI (Food Poverty Working Group 2022), with similar levels reported in Northern Ireland where 9% of households experience food insecurity (Northern Ireland Statistics and Research Agency 2025).

Both jurisdictions on the island of Ireland face multiple challenges that compound food insecurity. Persistent structural issues including wage stagnation, precarious employment, inadequate income, inadequate social welfare provision, and housing unaffordability have continued for years (Ipsos and The Trussell Trust 2023, Joseph Rowntree Foundation 2024). The COVID-19 pandemic (2020–2022) intensified these vulnerabilities, and subsequent inflation and cost-of-living pressures have further exacerbated food insecurity. Recent survey data from ROI (*n* = 1130 parents of children aged 0–18 years) found that 51% of respondents reduced spending on fuel and other essentials to afford food (Barnardos 2022). The ROI recorded the highest household expenditure costs in the European Union (EU) in 2023, with prices 46% above the EU average (Eurostat 2023). In NI, Brexit—the UK's withdrawal from the EU in 2020 has contributed to increased food prices through additional administrative requirements and trade barriers, with disproportionate effects on lower-income households who allocate a higher proportion of income to food purchases (Barons and Aspinall 2020, Caraher *et al*. 2023).

Government responses in both jurisdictions include a mix of statutory and voluntary sector initiatives. The ROI distributes Fund for European Aid to the Most Deprived (FEAD) resources through voluntary organisations for food assistance (Department of Social Protection 2023). NI has piloted Social Supermarket initiatives, which combine subsidised food access with wraparound support services (Department for Communities 2022). Both jurisdictions operate school meal programmes, with ROI committed to implementing universal free school meals by 2030, while NI maintains means-tested provision (Department of Education 2023). Both states have obligations under the International Covenant on Economic, Social and Cultural Rights regarding the right to adequate food (Caraher and Furey 2018).

### News media framing of food poverty and insecurity

Given the significant health impacts and prevalence of food poverty and insecurity, analysing how news media communicate this issue becomes increasingly important (Wells and Caraher 2014, Kerins *et al*. 2023, Kerrigan *et al*. 2025). News media play key roles in raising the profile of social and health-related issues and framing them in ways that shape how audiences interpret problems, assign responsibility for causes, and identify potential solutions (Entman 1993). With increased media attention to food poverty and insecurity in developed countries (Wells and Caraher 2014, Collins *et al*. 2016, Knight *et al*. 2018, Yau *et al*. 2021), these framings inform judgement and action among both citizens and policy makers (Matthes 2009, Kerrigan *et al*. 2025).

A recent rapid review examined news media framing of food poverty and insecurity in high-income countries (Kerins *et al*. 2023). The review identified 10 studies, predominantly of low methodological quality, all limited to newspaper analysis with most examining national rather than local print media. The findings revealed limited nuanced understanding of this issue in media coverage. Newspapers typically defined the problem through food bank usage and emphasized physical health consequences whilst neglecting broader dimensions. Although structural causes were frequently acknowledged, charitable solutions dominated the proposed responses. Government officials and charity representatives comprised the primary sources, whilst voices of those experiencing this issue remained largely absent.

Building on the review findings, recent analysis of broadcast media on the island of Ireland revealed distinct framing patterns (Kerrigan *et al*. 2025). The study found that broadcast media frequently defined food poverty and insecurity through food bank usage and rising cost-of-living, with particular emphasis on ‘holiday hunger’ affecting children. Coverage portrayed this issue as potentially affecting anyone, especially the ‘working poor’, while framing solutions predominantly through charitable responses despite acknowledging structural causes. Government inaction emerged as a key causal frame, particularly in NI where political instability was frequently cited. The study revealed important differences between media formats: television coverage often conveyed shame and stigma through visual representation strategies, while radio provided more nuanced discussions with greater inclusion of voices from those experiencing food poverty and insecurity.

While these broadcast media findings provide important insights, newspaper coverage on the island of Ireland remains unexamined. This represents a significant gap, as newspapers remain an influential news source (Peterson 2021) and examining their coverage is essential to develop a comprehensive understanding of media discourse on food poverty and insecurity. Responding to calls for methodologically rigorous studies and comparative analysis of national versus local news media coverage (Kerins *et al*. 2023), this study provides the first systematic examination of how newspapers frame food poverty and insecurity on the island of Ireland.

The current study employs Entman's (1993) framing theory as the *a priori* analytical framework. Entman conceptualizes news frames as selections that make ‘some aspects of a perceived reality more salient in a communicating text’ to promote ‘a particular problem definition, causal interpretation, moral evaluation, and/or treatment recommendation’ (p.52). This framework enables systematic examination of how newspapers construct meaning around this issue. The primary objective was to examine framing patterns across the island of Ireland, with secondary objectives comparing coverage between (1) jurisdictions (ROI and NI) and (2) national and local newspapers.

## METHODS

This study was conducted as part of a larger project exploring communication of food poverty on the island of Ireland, which aimed to investigate how the media portrays food poverty and to explore public, policy, and key stakeholder perceptions of the topic (Vaughan *et al*. 2024).

### Sampling of newspapers and articles

Newspaper articles were sampled using two databases. LexisNexis provided access to national and regional newspapers from both the ROI and NI. For regional and local newspapers not available in LexisNexis, a purposive sample was selected from the Irish Newspapers Archives to ensure geographic representation across all provinces (Table 1). Both databases were searched using the terms ‘food poverty’ and ‘food insecurity’ between 01 January 2018 and 01 January 2023. The rationale for not using other search words and phrases, such as ‘food banks’ and ‘holiday hunger’, was to avoid biasing the results in terms of print media framing of the definition of and solutions to food poverty. The search timeframe was chosen to capture contemporary print media framing, including coverage before, during and after the COVID-19 pandemic, the cost-of-living crisis/energy crisis and other geo-political events impacting income and food affordability, accessibility, and availability.

**Table 1.** Selected newspapers from each province of Ireland in the Irish Newspaper Archives.

Leinster	Munster	Connaught	Ulster
The Echo	Limerick Leader	Connacht Tribune	Belfast Newsletter
Meath Chronicle	Evening Echo	Connaught Telegraph	Tyrone Herald
Leinster Express	Tipperary Star	Leitrim Observer	Fermanagh Herald
Westmeath Examiner	Munster Express	Tuam Herald	Donegal Democrat
Kilkenny People	Clare Champion	City Tribune	Northern Standard
Dundalk Democrat	–	–	–

Note: Ireland comprises four provinces: Ulster, Leinster, Munster, and Connacht. Six of Ulster's nine counties form NI (part of the UK), while the remaining three Ulster counties and all other provinces are in the ROI.

### Article selection

Articles were downloaded and duplicates removed. Two researchers (C.K., T.J.) independently screened articles against inclusion and exclusion criteria (Table 2). Articles from national and regional/local newspapers were eligible if the main focus of the article was on food poverty or food insecurity in the context of the ROI and/or NI. Online-only publications were excluded to maintain consistency in media type analysed. Discrepancies in eligibility screening of articles were resolved through discussion.

**Table 2.** Article eligibility criteria.

	Inclusion criteria	Exclusion criteria
Article type	Features, reports, news articles, opinion pieces	Letters to editor, obituaries, classifieds (ads/notices)
Publication type	National and local/regional newspapers	Industry publications, magazines, online only publications
Topic focus	Articles that focus on food poverty/food insecurity in the context of the ROI and/or NI	Articles where food poverty/food insecurity is not the main focus (i.e. mentioned in passing) and/or not in the context of the ROI or NI
Word count	Equal to or greater than 250	Less than 250

### Data analysis

All included articles were imported into NVivo 14 software for data management. The first phase of analysis involved two researchers (C.K., T.J.) investigating patterns in publication frequency. The number of included articles was counted by newspaper title, territory (published in the ROI or NI), coverage (national or regional/local), category (broadsheet or tabloid), and article type (news story, feature article or opinion piece). Additionally, temporal patterns were examined by calculating monthly publication frequencies. Articles from peak coverage periods were analysed to identify themes associated with increased media attention.

In the second phase of analysis, a combination of deductive and inductive coding was performed with Entman's (1993) framing theory as the *a priori* framework. Entman's conceptualization of framing consists of the following elements: defining the problem, identifying causal interpretations, proposing treatment recommendations (solutions), and offering moral evaluations. Articles were systematically coded by the first author (C.K.) according to these four functions. To capture themes within Entman’s four framing functions, thematic analysis was performed, involving systematic coding of articles and grouping of similar codes into themes (Saldana 2021). To ensure reliability, a subset of articles (*n* = 8) was independently coded by two researchers (T.J., E.V.). Regular meetings were held between coders to discuss and refine the coding framework, ensuring consistency in code application. Finally, the frequency of themes within each framing function was calculated by newspaper territory (ROI, NI) and type (national, regional/local) to identify patterns and key differences in coverage.

## RESULTS

### Sample characteristics

Of the 823 articles screened, 80 (10%) met the eligibility criteria for inclusion (see Table 3). Over half the articles were from newspapers published in the ROI (60%, *n* = 48), with 40% (*n* = 32) from newspapers published in NI. Regional/local newspapers accounted for 65% (*n* = 52) of articles, with national newspapers representing 35% (*n* = 28). Most national newspapers were broadsheet (82%, *n* = 23), with tabloid newspapers accounting for 18% (*n* = 5). News stories comprised 55% (*n* = 44) of articles, feature articles 40% (*n* = 32), and opinion pieces 5% (*n* = 4). The *Belfast Telegraph* contributed the largest proportion of articles (28%, *n* = 22), followed by the *Irish Times* (14%, *n* = 11).

**Table 3.** Number of included articles by newspaper title, territory, coverage, and category.

Newspaper title	Number of articles (%)	Territory	Coverage	Category
*Belfast Telegraph*	22 (27)	NI	Regional	Broadsheet
*Bray People*	1 (1)	ROI	Regional	ND
*Corkman*	1 (1)	ROI	Regional	Tabloid
*Irish Daily Mail*	3 (4)	ROI	National	Tabloid
*Derry Journal*	10 (12)	NI	Regional	Tabloid
*Enniscorthy Guardian*	1 (1)	ROI	Regional	ND
*The Herald*	2 (2)	ROI	National	Tabloid
*Irish Examiner*	9 (12)	ROI	National	Broadsheet
*Irish Independent*	3 (4)	ROI	National	Broadsheet
*The Irish Times*	11 (14)	ROI	National	Broadsheet
*Limerick Leader*	1 (1)	ROI	Regional	Broadsheet
*The Echo/Evening Echo*	10 (12)	ROI	Regional	Tabloid
*Westmeath Examiner*	3 (4)	ROI	Regional	Broadsheet
*Wexford People*	3 (4)	ROI	Regional	Tabloid
**Total**	**80** (**100)**	–	–	–

ND, no data; NI, Northern Ireland; ROI, Republic of Ireland.

**Figure 1.**
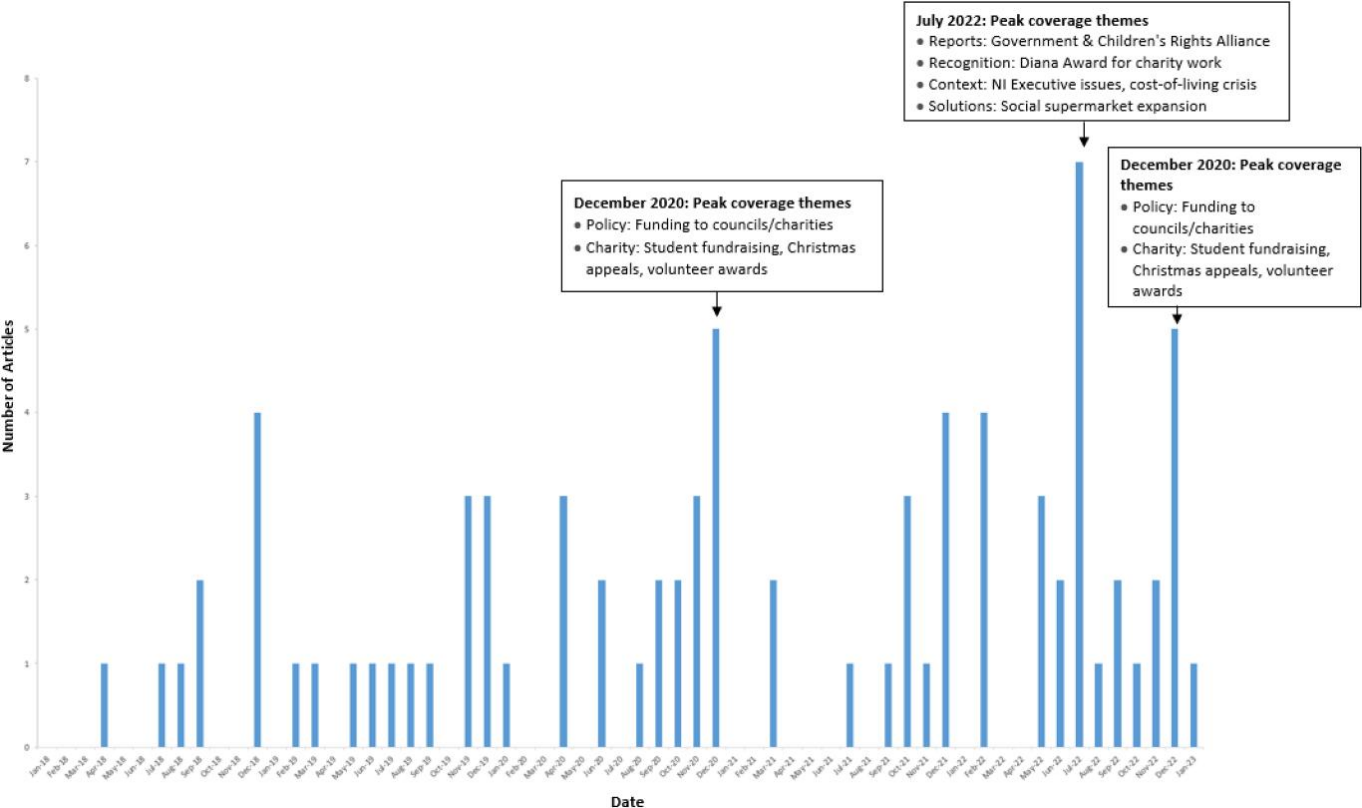
Number of articles on food poverty and insecurity by month, January 2018 to January 2023.

Article publication varied by year and month (see Fig. 1). The highest proportion of articles appeared in 2022 (34%, *n* = 27), followed by 2020 (24%, *n* = 19). December was the most frequent month of publication, accounting for 24% (*n* = 19) of articles. January had the lowest publication rate (1%, *n* = 1), with March, April, May, and August each contributing 5% (*n* = 4). July 2022 had the highest monthly total (*n* = 7), followed by December 2022 (*n* = 5) and December 2020 (*n* = 5).

## FRAMING OF FOOD POVERTY AND INSECURITY

The following sections present findings organized according to Entman's four framing functions: problem definition, causal interpretation, treatment recommendations (solutions), and moral evaluation. Key differences between territories (NI vs ROI) and newspaper types (national vs regional/local) are noted throughout. Frequencies for all themes within each framing function by newspaper territory and type are presented in Supplementary Table S1.

### Definitions of the problem of food poverty and insecurity

News articles portrayed food poverty and insecurity through multiple dimensions. The vast majority used the term ‘food poverty’ rather than ‘food insecurity’. Articles most frequently characterized the problem through insufficient food quantity and hunger, with national newspapers more likely to emphasize these aspects than regional/local newspapers. Some articles also referenced dietary quality, describing inability to access fresh produce or nutritionally adequate food.

‘*Then we have had children over time literally ravenous, literally taking it with their hands, they can't get enough of it into their mouths and can't get it in quick enough.’* (Chef at Barnardos quoted in the *Irish Examiner*, 23 February 2022)

Articles also identified hidden dimensions of food poverty and insecurity through concepts of ‘hidden hunger'—reflecting stigma and invisibility—and ‘holiday hunger’, highlighting children's vulnerability during school breaks. Food poverty and insecurity were commonly linked with charity usage, with articles frequently using food bank statistics and descriptions of food parcel distribution to illustrate the extent of the problem.

‘We wanted to give people a place where they could come to where the stigma of other people looking at them, seeing where they were going, was removed. No-one wants to have to go to a food bank, and it takes a lot of courage to come in and say you need help.’ (Manager of Hope Food Bank quoted in Belfast Telegraph, 12 February 2022)

‘The Trussell Trust, which operates a network of food banks throughout the UK, revealed only yesterday that the number of people in receipt of food parcels in Northern Ireland has increased by almost a third to 17 571 in the sixth months to September. Heartbreakingly, as many as 41% of these (7260) were children.’ (Belfast Telegraph, 14 November 2019)

Articles portrayed diverse consequences of food poverty and insecurity for individuals and families. Mental and social consequences appeared more frequently than physical health impacts, with coverage describing anxiety, shame, stigma, and social isolation. Additionally, articles described household coping strategies, particularly parents going without meals to ensure children were fed—a theme that appeared more frequently in national newspapers than regional/local publications.

‘People feel so ashamed and embarrassed even though they have done nothing wrong. They feel that they have let their families down.’ (Founder of a local food charity quoted in the Westmeath Examiner, 25 June 2022)

‘Some parents who we support tell me that they'll always make sure their children have a decent meal but that means the parent is going without a meal. They might have a sandwich instead.’ (National Policy Manager for Barnardos quoted in the Irish Times, 26 November 2022)

### Causal attributions of food poverty and insecurity

**Figure 2.**
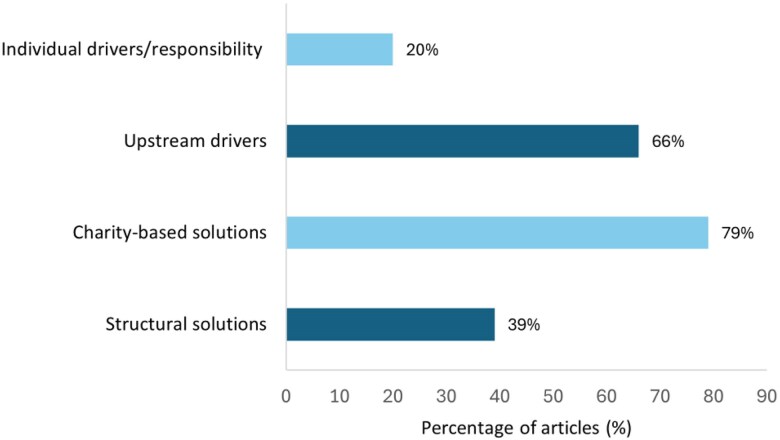
Disconnect between identified drivers and proposed solutions (*n* = 80 articles).

The majority of articles included causal explanations for food poverty and insecurity. Upstream structural factors appeared in 66% of articles compared to individual factors in 20% (Fig. 2). National newspapers were more likely to include causal interpretation than regional/local newspapers.

### Immediate structural drivers

Economic factors were the most frequently cited immediate drivers of food poverty and insecurity. Insufficient income appeared in over three-quarters of articles, with coverage patterns varying by region and newspaper type. Newspapers in the ROI emphasised high cost of living more frequently than NI publications, while also giving greater attention to low wages and unemployment, including how parents have been forced out of work by childcare costs. In contrast, NI newspapers provided more coverage of seasonal financial pressures, particularly Christmas costs.

‘I have three young children and I had to give up my job due to childcare costs. Ever since then, it has just been downhill and I'm finding myself struggling more and more as the weeks go on.’ (Member of public experiencing food poverty quoted in Westmeath Examiner, 25 February 2022)

‘Concerns have been raised at the number of children, young people and parents who will have no food on the table during the festive season amid the additional stress and pressure on parents of buying Christmas presents and toys.’ (Belfast Telegraph, 11 December 2019)

Issues of accessibility, including the specific barriers to food among those experiencing homelessness, appeared only in ROI newspapers, with no coverage in NI publications. Articles citing accessibility barriers, such as rural isolation and lack of transport, appeared predominantly in national newspapers compared to regional/local publications. Similarly, homelessness as a driver of food poverty and insecurity, including lack of cooking facilities and storage, appeared primarily in national newspaper coverage.

‘People living in rural areas without access to a car can't take advantage of deals and offers in the larger supermarkets and local convenience shops tend to be more expensive.’ (Head of Social Justice at SVP quoted in the Irish Times, 2 July 2018)

‘The scheme would benefit homeless families who did not have access to cooking facilities as well as low-income households struggling to make ends meet.’ (St Vincent de Paul's social policy development officer quoted in the Irish Times, 26 August 2019)

### Upstream drivers

Articles focusing on upstream drivers appeared more frequently in national newspapers and those published in the ROI. The COVID-19 pandemic and resulting economic fallout appeared as upstream drivers in approximately one-third of articles, with coverage more prevalent in ROI newspapers than NI publications. Articles described increased demand for food aid services and new demographics seeking help during the pandemic.

‘…Barnardos saw an increase in the number of families needing help during the pandemic. ‘We were experiencing people coming to us looking for help who had never come before.'’ (Chief Executive Officer of Barnardos quoted in the Irish Independent, 22 February 2022)

Governmental policies and welfare system issues were commonly cited as structural drivers, appearing more frequently in national newspapers than regional/local publications. Articles highlighted welfare system failures including inadequate benefit values, payment delays, benefit sanctions, and delays in COVID-19 pandemic payments. Additional governmental drivers included absence of (universal) school meals schemes, political instability in NI, and broader public service inadequacies, though these received less frequent coverage.

‘In the last two weeks of January, some families came to the centre on a Wednesday, the day before social welfare payments, as they had no money left and had to decide between heat or light or buying food’ (Associate Director of Children's Services at Barnardos quoted in the Irish Examiner, 23 February 2022)

‘The top three reasons for food poverty given by food bank users were low income, benefit changes and benefit delays.’ (Belfast Telegraph, 12 February 2022)

### Solutions to address food poverty and insecurity

Solutions reported in articles were predominantly charity-based, appearing in 79% of articles compared to structural solutions in 39%. National newspapers were more likely to include structural solutions than regional/local publications.

### Charity-based solutions

Existing solutions to food poverty and insecurity reported within articles were predominantly charity-based. Food banks were the most frequently mentioned charitable response, appearing more often in NI newspapers than ROI publications. NI coverage also gave greater attention to redistribution of food waste initiatives, where retailers redirect surplus food to charities, and social supermarkets offering discounted food access.

‘We opened Northern Ireland's first social supermarket in 2017 and through the charity FareShare, which accesses surplus stock from Tesco, Asda and Lidl, we can sell good, in-date food to our members at 50% to 70% below supermarket prices.’ (Chief Executive Officer at Footprints Women's Centre quoted in the Belfast Telegraph, 25 March 2021)

Over a third of articles reporting on charity-based solutions acknowledged the need for structural solutions to address root causes of food poverty, with this acknowledgment appearing more frequently in national newspapers than regional/local publications. Articles described charitable responses as providing immediate relief while questioning their long-term sustainability. Some articles also critiqued the charitable model as addressing symptoms rather than causes.

‘Food banks do all they can to help families in Northern Ireland over the summer… But no charity can replace the dignity of having enough money for the basics’ (Area Manager for The Trussell Trust in Northern Ireland quoted in the Belfast Telegraph, 27 June 2019)

‘…charity was one way of redressing unfair economic imbalances but said the fact poverty existed was a ‘government problem’ that needed to be addressed.’ (Social work students quoted in Derry Journal, 30 December 2020)

### Structural solutions

Structural solutions to food poverty were proposed primarily through policy interventions. Free school and holiday meals programmes were the most frequently mentioned structural solution, appearing in nearly one-third of national newspapers but only 12% of regional/local publications. Articles presented these programmes as both existing interventions and proposed expansions.

‘More than 100 000 children in Northern Ireland benefited from an extension of free school meals over the summer… Education Minister Peter Weir said 100 570 children received assistance costing £11 942 445.’ (Belfast Telegraph, 28 September 2020)

‘We know the school meals programme is one effective way of reaching very vulnerable children… We are calling for its extension through the summer months to help support families who are bearing the weight of this public health crisis and, ultimately, prevent children going hungry. Holiday hunger isn't acceptable.’ (Chief Executive of the Children's Rights Alliance quoted in the Irish Times, 11 June 2020)

Welfare reform appeared less frequently, though still showed higher coverage in national newspapers. Articles called for various reforms including adjusting payments to match living costs, addressing payment delays, and expanding eligibility.

‘Much progress can be made through the introduction of a living wage, income security and a responsive, adequately funded welfare system.’ (Academic from University College Dublin quoted in Irish Examiner, 01 June 2022)

### Moral evaluation of food poverty and insecurity

Descriptions of those experiencing food poverty and insecurity as deserving of assistance appeared frequently in coverage, with national newspapers more likely to include such descriptions than regional/local publications. Articles described those affected as facing circumstances beyond their control, particularly the COVID-19 pandemic and cost-of-living crisis. In contrast, descriptions of food aid recipients as undeserving appeared rarely across coverage.

‘We have young people here trying to get a mortgage, trying to get married and they can't afford it, as the rent is so high, so they can't get a mortgage. Every family, every person, has the right to have a decent home, food and respect.’ (Founder of the Capuchin Day Centre quoted in the Irish Independent, 30 December 2019)

Coverage emphasised families with children and employed individuals when describing those seeking support. Articles frequently used terms such as ‘working poor’ and ‘people like us’, highlighting food poverty and insecurity among essential workers including nurses.

‘As a nurse who lives in the midlands, I can tell you that nurses are not immune to food poverty. At least six nurses are receiving assistance from food banks. This is shocking in 2022.’ (General Nurse at Tullamore Hospital quoted in the Irish Independent, 7 May 2022)

‘I am amazed at some of the people coming forward. You'd never dream they would need help. In their own communities they would be seen as well to do, but they don't have two cents to rub together.’ (Founder of a local food poverty charity quoted in the Westmeath Examiner, 25 June 2022)

Positive descriptions of donors and volunteers appeared in 28% of articles overall, with higher coverage in NI newspapers (41%) compared to ROI publications (19%). Regional/local newspapers included these positive descriptions more frequently than national publications. Articles used terms such as ‘selfless,’ ‘compassionate,’ and ‘noble’ when describing volunteers and donors.

‘…the virtual army of volunteers who nobly give up their time to look out for those who are worse off.’ (Belfast Telegraph, 14 November 2019)

‘The Diana Award was established with a core aim of recognising extraordinary young people who are selflessly leading positive social change, and Ben could not be more deserving of this accolade.’ (Chief Executive of the Diana Award quoted in the Belfast Telegraph, 24 December 2020)

### Social actors involved in the discourse

Charities appeared as the dominant voice in coverage of food poverty and insecurity, featured in 88% of articles. Government and policy officials appeared in approximately half of articles, with greater representation in national newspapers (75%) than regional/local publications (40%). The private sector, including supermarket chains and banks, appeared in 25% of articles. Notably, only 10% (*n* = 8) of articles included direct voices from those experiencing food poverty and insecurity, with charities frequently serving as proxy voices speaking on behalf of affected individuals.

## DISCUSSION

The primary objective of this study was to examine framing patterns across the island of Ireland, with secondary objectives comparing coverage between (1) jurisdictions (ROI and NI) and (2) national and local newspapers. This discussion first interprets the differences observed between jurisdictions and between national and local newspaper coverage, then critically examines two key aspects of the framing of food poverty and insecurity: the use of food banks as a proxy for the issue, and the spotlighting of downstream solutions to upstream problems.

### High-level overview and interpretation of reporting patterns

Reflecting similar trends in the UK (Yau *et al*. 2021), analysis of a sample of newspaper articles from the island of Ireland suggests that reporting of food poverty and insecurity increased between 2018 and 2023, with a particular uptick during and after the COVID-19 pandemic. For the most part, reporting appeared episodic, revolving around specific events. July 2022 saw the highest monthly coverage, while December consistently showed annual peaks, suggesting media attention to food poverty and insecurity intensifies during school holiday periods—both summer and Christmas—when ‘holiday hunger’ becomes salient. This pattern of tethering food poverty and insecurity reporting to holiday periods aligns with previous analysis of broadcast media coverage in Ireland (Kerrigan *et al*. 2025) and patterns identified in UK newspaper coverage (Yau *et al*. 2021). The limited inclusion of voices from those experiencing food poverty and insecurity—appearing in only 10% of articles with charities frequently serving as proxy voices—reflects patterns documented in UK studies (Wells and Caraher 2014, Knight *et al*. 2018).

Proportionally, there was greater coverage of food poverty and insecurity in the media in NI, with 40% of the total sample emanating from just two Northern Irish papers—the Belfast Telegraph and the Derry Journal. While it is unclear why proportionally there was more reporting of the topic in NI as opposed to the ROI, comparable research exploring differences in reporting on food and fuel poverty in the UK showed that Scottish newspapers accounted for 30% of that sample (*n* = 185), with the authors suggesting that the issue may be of greater interest to the northerly region due to a combination of factors including location and climate, rurality, smaller population, and greater levels of social and economic inequality compared to their neighbours in England (Champagne *et al*. 2024, de Haro Moro *et al*. 2025). While direct economic comparisons between NI and the ROI are complicated as a result of their structural differences, data suggest that the Republic outperforms the North across a range of relevant metrics for living standards and income; for example, household disposable income is 18.3% higher in the Republic as compared to the North, while hourly wages are 36% higher (Bergin *et al*. 2025).

### Local vs national reporting as operations of ideology and social practice

Differences were noted also along national and regional/local lines in terms of focus and tone of reporting, with the former tending towards greater analysis of causal factors, policy issues, and structural solutions, and the latter tending towards a greater focus on and praise for the work of charities and community food aid initiatives. These findings are perhaps unsurprising, and speak to the distinct social practices and functions of local and national papers. Studies in the US, for example, that have explored differences between local and national framing of news stories on disparate topics have found that national outlets are more likely to emphasize the wider social and policy implications, while local media tend to take a human-interest slant and focus on concerns more immediate to the local community (Holt and Major 2010, Holody and Daniel 2017).

Likewise, slight albeit key differences were apparent between the two jurisdictions, further reflective of the national and regional statuses of the ROI and NI, respectively. These included a greater emphasis in the ROI on structural and economic factors, accessibility issues, and the specific impact on those experiencing homelessness, while food banks, food redistribution initiatives, and positive descriptions of volunteers were a more frequent feature of reporting in NI. These divergences across both dimensions—national/local newspaper type and the national/regional political status of ROI/NI—may reflect differences in proximity to audience and newspaper ideology. Bros and Gatica-Perez (2025), for example, have suggested that local outlets prioritize concepts such as shared local identity, sense of community belonging, and relevance to the daily lives of their readers, while national papers position themselves in an authoritative and interpretive role as an intermediary between the complexities of social and political life and the wider public.

### Food banks as a proxy for food poverty and insecurity

Food poverty and insecurity were frequently framed around or tethered to the concept of food bank usage, reflecting a common trend in reporting on this issue in the UK (Wells and Caraher 2014, Knight *et al*. 2018, Yau *et al*. 2021) and other high-income countries (Kerins *et al*. 2023). While the trope of the food bank may be a useful signifier for journalists in telling the story, it is likely this means the whole story is not being told. For one, there is evidence to suggest food bank usage may be a poor proxy for measuring food poverty and insecurity. For instance, in NI 9% of households experience food insecurity (Northern Ireland Statistics and Research Agency 2025) while UK data show only 3.6% access food aid (Department for Work and Pensions 2025). This suggests that the issue may be under-reported in the media, thus obscuring its scale and the associated health impacts.

More saliently perhaps from a social justice—and health promotion—perspective, is how the discursive tethering of food banks to the issue of food poverty and insecurity belies the ineffectiveness and unsustainability of this method of addressing the problem. For instance, Caraher and Davison's (2023) critique of the food bank and redistribution model characterizes the approach as a ‘successful failure’, which results in food waste regardless through inappropriate and out-of-date donations, and one in which nutritional inadequacy, instability, insufficiency, and indignity are embedded for those obliged to use it. While food banks may provide essential emergency relief, media focus on them as the primary solution obscures the need for systemic change. Alternative approaches that might better address this issue include the ‘cash first’ model advocated by organizations such as the Independent Food Aid Network in the UK and the ‘Good Food Area’ social enterprise model proposed by Caraher and Davison (2023), which adopts a social justice and direct farm-to-fork approach that minimizes food waste while empowering communities and ensuring the dignity of individuals.

### Downstream solutions to upstream problems?

That food aid and charitable solutions, including social supermarkets and food redistribution efforts, were touted as the primary means of addressing the problem of food poverty and insecurity becomes all the more illogical, given that analysis showed that reporting largely acknowledged the structural and policy underpinnings of the problem. This discordant tendency to propose ‘downstream solutions to upstream problems’ mirrors patterns identified in newspapers in the UK (Wells and Caraher 2014, Knight *et al*. 2018, Yau *et al*. 2021) and other high-income countries (Kerins *et al*. 2023), with similar patterns documented in broadcast media on the island of Ireland (Kerrigan *et al*. 2025).

The legitimization of downstream solutions in media discourse should, however, be viewed with alarm by health promoters, not just for symbolically endorsing the concept of food aid as a valid solution, but for the de facto way in which it obscures the root causes of entrenched social, income and health inequalities. The effect of such representations is to embed the perception of the issue as natural, and food aid as a ‘common sense’ measure, contributing to what sociologist Pierre Bourdieu termed as *doxa*, or the [mis]recognition of social inequality as taken-for-granted, rather than as an arbitrary state of affairs that arises out of decision-making grounded in political, socioeconomic, and ideological orthodoxy (Bourdieu 1977, 1991). Tacit acceptance of this issue as an inevitable outcome, and food aid as reasonable solution thereof, also lessens the moral imperative of governments, as duty-bearers, to ensure the rights of their citizens to adequate food are protected, respected and fulfilled, while further giving comfort to the private sector, who might otherwise be called upon to ensure salaries are adequate to meet people's needs (Furey 2025). Thus, reframing of food poverty and insecurity through a rights-based and public health lens is needed to tilt the focus towards approaches that promote health, justice, and dignity for all.

### Implications for health promotion practice

The disconnect between acknowledged structural causes and proposed charitable solutions identified in newspaper coverage presents fundamental challenges for health promotion practice. Our analysis revealed that whilst newspapers recognize systemic drivers of food poverty and insecurity, they predominantly frame solutions through charitable responses. This misalignment requires strategic interventions from health promotion practitioners.

First, health promotion advocacy must challenge the normalization of both the problem and its supposed solutions. Our analysis demonstrated how media coverage contributes to what Bourdieu termed doxa—the misrecognition of social inequality as natural and unchangeable. The legitimization of food banks as commonsense responses obscures state responsibility whilst enabling the persistence of the structural failures that drive food poverty and insecurity. Health promoters should therefore develop counter-narratives that position food poverty and insecurity in wealthy nations as evidence of policy failure requiring systemic reform, not charitable intervention.

Second, the episodic nature of coverage—with December peaks and focus on ‘holiday hunger’—presents both challenges and opportunities. Health promotion practitioners should maintain consistent messaging about systemic causes throughout the year whilst strategically using December coverage to shift focus from charitable appeals to policy reforms. This requires evidence-based resources that can quickly reframe seasonal charity narratives towards sustainable solutions when media attention peaks.

Third, the marked differences between national and local newspaper coverage identified in our analysis necessitate targeted communication approaches. Our findings revealed that whilst national newspapers demonstrated engagement with systemic analysis and policy solutions, local newspapers prioritized charitable responses and community initiatives. This divergence indicates that health promotion practitioners should prioritize engagement with local media through strategies that align with local reporting cultures. This includes partnering with community groups to develop and promote alternative models such as ‘cash first’ approaches and social enterprise initiatives like the ‘Good Food Area’. Health promoters can support journalists in presenting these initiatives as community-based alternatives that address food poverty and insecurity through financial autonomy and local food systems rather than charitable dependency.

These recommendations position health promotion practitioners as advocates who challenge the normalization of both the problem and its charitable responses. By consistently linking structural problems to policy solutions rather than charitable interventions, health promoters can shift public discourse from managing symptoms to addressing causes, ultimately advancing the realization of the right to adequate food.

### Strengths and limitations

This study provides the first systematic examination of how food poverty and insecurity were portrayed in newspapers across the island of Ireland from January 2018 to January 2023, a period encompassing significant social and economic disruption including the COVID-19 pandemic and cost-of-living crisis. Unlike previous studies that focused on single countries or regions, we conducted a comparative analysis between the ROI and NI, revealing important differences in framing patterns between these contexts. Furthermore, we compared coverage between national and regional/local newspapers, addressing calls for such comparative analysis (Kerins *et al*. 2023). The use of Entman's (1993) established framing theory enabled systematic examination of problem definitions, causal interpretations, solutions, moral evaluations, and social actors across our sample.

Despite these strengths, limitations should be acknowledged. Our search strategy employed only the terms ‘food poverty’ and ‘food insecurity’. While this approach was intended to minimize bias towards particular framings of the issue, it may have reduced our sample size by excluding articles that discussed these phenomena using alternative terminology. We were also unable to analyse the influence of newspaper political orientation on framing patterns due to the lack of available data for newspapers specific to the ROI or NI. Additionally, our analysis focused exclusively on print newspaper coverage and did not examine online-only articles, social media discourse, or other news sources. Given the rapid evolution of media consumption patterns, this focus on traditional print media may not fully capture contemporary discourse around food poverty and insecurity.

## CONCLUSION

This study provides the first systematic examination of newspaper framing of food poverty and insecurity on the island of Ireland. Our analysis reveals a fundamental disconnect: whilst newspapers acknowledge structural causes—political, economic, and social dysfunction—they predominantly frame solutions through charitable responses. This misalignment between problem identification and proposed solutions perpetuates what Bourdieu termed doxa, naturalizing both the widespread problem and inadequate charitable responses as inevitable rather than consequences of political choices.

Coverage varied by time period, jurisdiction, and newspaper type. Articles peaked during December and summer holidays, reinforcing episodic rather than sustained attention. Jurisdictionally, NI showed proportionally greater coverage, emphasizing seasonal financial pressures and charitable solutions including food banks and redistribution initiatives, whilst publications in the ROI focused more on structural economic factors and COVID-19 impacts. Across newspaper types, national publications demonstrated greater analysis of systemic drivers and policy solutions whilst local newspapers focused predominantly on charitable responses and community initiatives.

These findings have implications beyond media representation. By positioning food banks and other charitable responses as both proxy for and solution to food poverty, newspapers obscure the issue's true scale and legitimize responses that fail to address root causes. This framing enables continuation of structural failures whilst shifting state responsibility to voluntary sectors, undermining recognition of food as a fundamental right requiring government accountability.

Addressing this disconnect requires fundamental shifts in how food poverty and insecurity is framed. Recognition of the right to adequate food—enshrined in international law—establishes state obligation rather than voluntary charity. When media coverage aligns solutions with identified causes, for example, connecting inadequate wages to necessary policy reforms rather than charitable responses, it challenges current framings. Without such alignment, the naturalization of both food poverty and insecurity and charitable responses persists, obscuring how this problem in wealthy nations results from political choices that media coverage documents yet rarely challenges.

## Supplementary Material

daaf206_Supplementary_Data

## Data Availability

The data underlying this article were derived from publicly available newspaper articles accessed through LexisNexis and Irish Newspaper Archives databases.
