# Supplementary material for: Newspaper framing of food poverty and insecurity on the island of Ireland
Source: Health Promot Int. 2025 Nov 27;40(6):daaf206. doi: 10.1093/heapro/daaf206 (PMC12659789; doi:10.1093/heapro/daaf206)
Supplement: daaf206_Supplementary_Data [file daaf206_supplementary_data.docx]

**Supplementary Table S1. Frequencies for all themes within each framing function by newspaper territory and type**

|  |  | **By Newspaper Territory** | | **By Newspaper Type** | |
| --- | --- | --- | --- | --- | --- |
| **Entman’s Framing Function** | **Total articles**  **(*n*=80)** | **Northern Ireland (*n*=32)** | **Republic of Ireland (*n*=48)** | **National**  **(*n*=28)** | **Regional/Local**  **(*n*=52)** |
|  | ***n* (%)** | ***n* (%)** | ***n* (%)** | ***n* (%)** | ***n* (%)** |
| **Problem Definition** | **80 (100)** | **32 (100)** | **48 (100)** | **28 (100)** | **52 (100)** |
| Definition | 80 (100) | 32 (100) | 48 (100) | 28 (100) | 52 (100) |
| ‘Food poverty' term used | 77 (96) | 32 (100) | 45 (94) | 28 (100) | 49 (94) |
| Charity usage synonymous with food poverty | 64 (80) | 23 (72) | 41 (85) | 22 (79) | 42 (81) |
| Inadequate food quantity | 49 (61) | 19 (59) | 30 (63) | 23 (82) | 26 (50) |
| Hunger | 26 (33) | 9 (28) | 17 (35) | 10 (36) | 16 (31) |
| Skipping meals or reduced portions | 9 (11) | 0 (0) | 9 (19) | 7 (25) | 2 (4) |
| Poor dietary quality | 23 (29) | 9 (28) | 14 (29) | 11 (39) | 12 (23) |
| Illustrates poverty and deprivation | 18 (23) | 6 (19) | 12 (25) | 7 (25) | 11 (21) |
| ‘Food insecurity' term used | 5 (6) | 3 (9) | 2 (4) | 1 (4) | 4 (8) |
| Less socially acceptable food choices | 2 (3) | 0 (0) | 2 (4) | 2 (7) | 0 (0) |
| Multidimensional problem | 2 (3) | 0 (0) | 2 (4) | 1 (4) | 1 (2) |
| Consequences | 40 (50) | 15 (47) | 25 (52) | 13 (46) | 27 (52) |
| Mental and social consequences | 33 (41) | 10 (31) | 23 (48) | 12 (43) | 21 (40) |
| Physical health consequences | 12 (15) | 6 (19) | 6 (13) | 4 (14) | 8 (15) |
| Families distributing scare food | 11 (14) | 3 (9) | 8 (17) | 7 (25) | 4 (8) |
| Educational underachievement for children | 5 (6) | 2 (6) | 3 (6) | 1 (4) | 4 (8) |
| Poor health outcomes | 1 (1) | 0 (0) | 1 (2) | 1 (4) | 0 (0) |
| Unsafe feeding methods for babies | 1 (1) | 1 (3) | 0 (0) | 0 (0) | 1 (2) |
| **Causal Interpretation** | **75 (94)** | **27 (84)** | **48 (100)** | **28 (100)** | **47 (90)** |
| Immediate drivers | 65 (81) | 22 (69) | 43 (90) | 25 (89) | 40 (77) |
| Insufficient income | 62 (78) | 22 (69) | 40 (83) | 23 (82) | 39 (75) |
| High cost-of-living | 34 (43) | 8 (25) | 26 (54) | 16 (57) | 18 (35) |
| Low wages | 12 (15) | 1 (3) | 11 (23) | 10 (36) | 2 (4) |
| Unemployment / not working | 11 (14) | 1 (3) | 10 (21) | 6 (21) | 5 (10) |
| Christmas costs | 10 (13) | 6 (19) | 4 (8) | 1 (4) | 9 (17) |
| Unstable incomes | 9 (11) | 3 (9) | 6 (13) | 3 (11) | 6 (12) |
| Household size/structure | 6 (8) | 0 (0) | 6 (13) | 4 (14) | 2 (4) |
| Lone parent families | 4 (5) | 1 (3) | 3 (6) | 2 (7) | 2 (4) |
| Back to school costs | 2 (3) | 0 (0) | 2 (4) | 0 (0) | 2 (4) |
| Issues of accessibility to food | 7 (9) | 0 (0) | 7 (15) | 6 (21) | 1 (2) |
| Homeless problem | 6 (8) | 0 (0) | 6 (13) | 4 (14) | 2 (4) |
| School and service closure | 5 (6) | 0 (0) | 5 (10) | 3 (11) | 2 (4) |
| Domestic abuse | 2 (3) | 1 (3) | 1 (2) | 1 (4) | 1 (2) |
| Food chain supply issues | 1 (1) | 0 (0) | 1 (2) | 1 (4) | 0 (0) |
| Poverty | 1 (1) | 0 (0) | 1 (2) | 0 (0) | 1 (2) |
| Upstream drivers | 53 (66) | 16 (50) | 37 (77) | 23 (82) | 30 (58) |
| Covid-19 pandemic | 28 (35) | 8 (25) | 20 (42) | 12 (43) | 16 (31) |
| Governmental drivers | 25 (31) | 10 (31) | 15 (31) | 13 (46) | 12 (23) |
| Issues with welfare system | 14 (18) | 4 (13) | 10 (21) | 8 (29) | 6 (12) |
| Absence of school meals scheme | 6 (8) | 3 (9) | 3 (6) | 3 (11) | 3 (6) |
| Austerity measures | 3 (4) | 2 (6) | 1 (2) | 1 (4) | 2 (4) |
| No functioning Executive in Northern Ireland | 3 (4) | 3 (9) | 0 (0) | 0 (0) | 3 (6) |
| Inadequate public services | 1 (1) | 0 (0) | 1 (2) | 1 (4) | 0 (0) |
| Underfunding of third level education | 1 (1) | 0 (0) | 1 (2) | 1 (4) | 0 (0) |
| Individual drivers/responsibility | 16 (20) | 2 (6) | 14 (29) | 5 (18) | 11 (21) |
| Disability or illness | 5 (6) | 1 (3) | 4 (8) | 2 (7) | 3 (6) |
| Debt | 4 (5) | 1 (3) | 4 (8) | 0 (0) | 4 (8) |
| Substance abuse or addiction | 3 (4) | 0 (0) | 3 (6) | 1 (4) | 2 (4) |
| Lack of cooking skills | 2 (3) | 0 (0) | 2 (4) | 2 (7) | 0 (0) |
| Low literacy skills | 1 (1) | 0 (0) | 1 (2) | 1 (4) | 0 (0) |
| Care responsibilities | 1 (1) | 0 (0) | 1 (2) | 1 (4) | 0 (0) |
| Poor mental health | 1 (1) | 0 (0) | 1 (2) | 0 (0) | 1 (2) |
| Housing insecurity | 8 (10) | 0 (0) | 8 (17) | 6 (21) | 2 (4) |
| Inflation | 6 (8) | 0 (0) | 5 (10) | 2 (7) | 3 (6) |
| Forced migration | 4 (5) | 3 (9) | 1 (2) | 1 (4) | 3 (6) |
| **Treatment Recommendations** | **80 (100)** | **32 (100)** | **48 (100)** | **27 (96)** | **52 (100)** |
| Charity-based solutions^a^ | 63 (79) | 23 (72) | 40 (83) | 21 (75) | 42 (81) |
| Food banks | 31 (39) | 14 (44) | 17 (35) | 8 (29) | 23 (44) |
| Redistribution of food waste initiatives | 15 (19) | 11 (34) | 4 (8) | 2 (7) | 13 (25) |
| Social supermarket pilot programme or social grocery | 8 (10) | 7 (22) | 1 (2) | 0 (0) | 8 (15) |
| Other charity solutions | 15 (19) | 2 (6) | 13 (27) | 7 (25) | 8 (15) |
| Charity-based solutions but acknowledge need for structural solutions | 23 (29) | 8 (25 | 15 (31) | 14 (50) | 9 (17) |
| Structural solutions^a,b^ | 31 (39) | 11 (34) | 20 (42) | 17 (61) | 14 (27) |
| Free school and holiday meals | 15 (19) | 4 (13) | 11 (23) | 9 (32) | 6 (12) |
| Welfare reform | 7 (9) | 2 (6) | 5 (10) | 4 (14) | 3 (6) |
| Policies to tackle poverty | 5 (6) | 3 (9) | 2 (4) | 1 (4) | 4 (8) |
| Other structural solutions | 9 (11) | 2 (6) | 7 (15) | 6 (21) | 3 (6) |
| Solutions targeted at individuals^a,b^ | 12 (15) | 5 (16) | 7 (15) | 6 (21) | 6 (12) |
| Skills-based | 11 (14) | 6 (19) | 5 (10) | 5 (18) | 6 (12) |
| Knowledge-based | 1 (1) | 0 (0) | 1 (2) | 0 (0) | 1 (2) |
| Activism and raising awareness of food poverty^b^ | 6 (8) | 4 (13) | 2 (4) | 1 (4) | 5 (10) |
| Enhancing interagency collaboration^b^ | 2 (3) | 0 (0) | 2 (4) | 1 (4) | 1 (2) |
| Research to guide better decision and target resources^b^ | 1 (1) | 0 (0) | 1 (2) | 0 (0) | 1 (2) |
| **Moral Evaluation** | **79 (99)** | **31 (97)** | **48 (100)** | **28 (100)** | **51 (98)** |
| Deserving of assistance – desperate situations | 37 (46) | 15 (47) | 22 (46) | 15 (54) | 22 (42) |
| A sense of shock or outrage | 23 (29) | 11 (34) | 12 (25) | 9 (32) | 14 (27) |
| Positive attributes of fundraisers or donors | 22 (28) | 13 (41) | 9 (19) | 3 (11) | 19 (37) |
| Stark choices in how to spend money | 19 (24) | 5 (16) | 14 (29) | 11 (39) | 8 (15) |
| Calls for donation and volunteers | 14 (18) | 5 (16) | 9 (19) | 2 (7) | 12 (23) |
| Working poor - no body immune | 12 (15) | 1 (3) | 11 (23) | 7 (25) | 5 (10) |
| Challenges faced by food banks or other charities | 11 (14) | 2 (6) | 9 (19) | 3 (11) | 8 (15) |
| Rewards for volunteers or fundraisers | 11 (14) | 7 (22) | 4 (8) | 2 (7) | 9 (17) |
| Critique of food charity | 10 (13) | 7 (22) | 3 (6) | 3 (11) | 7 (13) |
| Volunteers framed as saviour/rescuer of 'poor' people | 9 (11) | 6 (19) | 3 (6) | 1 (4) | 8 (15) |
| Right to food and dignity | 9 (11) | 3 (9) | 6 (13) | 5 (18) | 4 (8) |
| Expectations of gratitude from food charity recipients | 7 (9) | 5 (16) | 2 (4) | 0 (0) | 7 (13) |
| Frustration with government lack of action | 4 (5) | 3 (9) | 1 (2) | 0 (0) | 4 (8) |
| Praise for food redistribution initiatives | 3 (4) | 1 (3) | 2 (4) | 1 (4) | 2 (4) |
| Food aid recipients framed as underserving | 2 (3) | 0 (0) | 2 (4) | 1 (4) | 1 (2) |
| Charitable food aid as a lifeline | 1 (1) | 0 (0) | 1 (2) | 1 (4) | 0 (0) |
| **Actors** | **80 (100)** | **32 (100)** | **48 (100)** | **28 (100)** | **52 (100)** |
| Charities | 70 (88) | 28 (88) | 42 (88) | 22 (79) | 48 (92) |
| Government/policy officials | 42 (53) | 16 (50) | 26 (54) | 21 (75) | 21 (40) |
| Private sectors | 20 (25) | 6 (19) | 14 (29) | 9 (32) | 11 (21) |
| Other actors/voices | 40 (50) | 21 (66) | 23 (48) | 18 (64) | 26 (50) |

*^a^ Existing solutions to food poverty*

*^b^* *Proposed solutions to food poverty*
